# Inpatient Costs of Treating Patients With COVID-19

**DOI:** 10.1001/jamanetworkopen.2023.50145

**Published:** 2024-01-03

**Authors:** Kandice A. Kapinos, Richard M. Peters, Robert E. Murphy, Samuel F. Hohmann, Ankita Podichetty, Raymond S. Greenberg

**Affiliations:** 1 RAND Corporation, Arlington, Virginia; 2 Department of Population Health, Dell Medical School, The University of Texas at Austin; 3 School of Biomedical Informatics, The University of Texas Health Science Center at Houston; 4 Vizient, Chicago, Illinois; 5 Peter O'Donnell Jr. School of Public Health, The University of Texas Southwestern Medical Center, Dallas

## Abstract

**Importance:**

With more than 6.2 million hospitalizations due to COVID-19 in the US, recognition of the average hospital costs to provide inpatient care during the pandemic is necessary to understanding the national medical resource use and improving public health readiness and related policies.

**Objective:**

To examine the mean cost to provide inpatient care to treat COVID-19 and how it varied through the pandemic waves and by important sociodemographic patient characteristics.

**Design, Setting, and Participants:**

This cross-sectional study used inpatient-level data from March 1, 2020, to March 31, 2022, extracted from a repository of clinical, administrative, and financial information covering 97% of academic medical centers across the US.

**Main Outcomes and Measures:**

Cost to produce care for each stay was calculated using direct hospital costs to provide care adjusted for geographic differences in labor costs using area wage indices.

**Results:**

The sample included 1 333 404 stays with a primary or secondary COVID-19 diagnosis from 841 hospitals. The cohort included 692 550 (52%) men, with mean (SD) age of 59.2 (17.5) years. The adjusted mean cost of an inpatient stay was $11 275 (95% CI, $11 252-$11 297) overall, increasing from $10 394 (95% CI, $10 228-$10 559) at the end of March 2020 to $13 072 (95% CI, $12 528-$13 617) by the end of March 2022. Patients with specific comorbidities had significantly higher mean costs than their counterparts: those with obesity incurred an additional $2924 in inpatient stay costs, and those with coagulation deficiency incurred an additional $3017 in inpatient stay costs. Stays during which the patient required extracorporeal membrane oxygenation (ECMO) had an adjusted mean cost of $36 484 (95% CI, $34 685-$38 284).

**Conclusions and Relevance:**

In this cross-sectional study, an adjusted mean hospital cost to provide care for patients with COVID-19 increased more than 5 times the rate of medical inflation overall. This appeared to be explained partly by changes in the use of ECMO, which increased over time.

## Key Points

**Question:**

How did the mean national hospital cost to treat patients with COVID-19 in inpatient settings change over the course of the pandemic?

**Findings:**

In this cross-sectional study of more than 1.3 million inpatient stays across the US, the adjusted direct cost to provide treatment increased from $10 394 at the end of March 2020 to $13 072 by March 2022, on average, adjusting for patient, stay, and hospital-level characteristics. Significant heterogeneity in costs by the comorbid conditions across US geographic regions and by patient discharge status were observed.

**Meaning:**

The findings of this study suggest that average hospital cost to provide inpatient treatment during the largest pandemic in more than 100 years in the US increased 26% over a 2-year period; costs to provide inpatient care increased even as care practices changed, vaccination rates increased, and the variants of concern evolved.

## Teaser

This cross-sectional study examines changes in hospital costs for inpatient stays of patients with COVID-19 across the US.

## Introduction

The COVID-19 pandemic placed unprecedented demands on medical services worldwide. Cumulative estimates of cases exceeded 660 million cases worldwide with nearly 6.7 million deaths through the end of 2022.^1^ One in 7 of these events occurred in the US (15.3% of cases and 16.3% of deaths) despite having only 4.25% of the world’s population.^1^ The peak demand for US hospital services occurred during the Omicron variant surge (November 2021 through February 2022), when patients with COVID-19 accounted for more than one-fifth of hospital admissions and nearly one-third of intensive care unit (ICU) beds.^1^ The surges in demand for COVID-19 inpatient treatment resulted in canceled elective surgeries^2^ with accompanying loss of revenue^3^; increased ICU capacity^4^; shortages of personnel, medications, and equipment^5^; and diminished hospital operating margins.^6^

Despite the overwhelming financial impact of COVID-19 on hospitals in the US, relatively little has been published on hospital costs to deliver inpatient care to an unprecedented number of patients. For example, there were 2.38 million new admissions of patients with confirmed COVID-19 in the 2020-2021 season^7^ vs 380 000 hospitalizations due to influenza in 2018-2019.^8^ Most studies to date have focused primarily on the early phases of the pandemic or have used payment rates as a proxy for costs. There have been 3 studies^9,10,11^ analyzing cost data using the Premier Healthcare Database, which includes approximately 10 million inpatient admissions per year from more than 800 hospitals,^12^ The first 2 studies analyzed inpatient costs of stays in 2020 and reported median costs of $11 267 and $12 046.^9,10^ A more recent study used the same file, extending analyses through July 2021 and aggregating multiple stays across patients, reporting an unadjusted mean cost of all hospitalizations per patient at $24 826.^11^ In addition, studies examining Medicare beneficiaries reported average payments ranging from $21 752 to $24 033 per stay,^13^ but third-party payments do not necessarily reflect the financial burden patients experience from out-of-pocket expenses, the hospital and health professional costs to deliver care, or the amount of medical resources used.^13,14,15^

Evidence to date has also documented certain chronic conditions as risk factors for more severe disease and greater risk of hospitalization and mortality among patients contracting COVID-19, including obesity, diabetes, chronic lung diseases, and kidney diseases.^16,17,18,19,20^ The extent to which the presence of these conditions translated into greater health care treatment use and costs, however, has not been well documented. This study’s aim is to contribute to the evidence on the mean hospital cost to provide inpatient care for patients with COVID-19 and how it varied through the pandemic and by important sociodemographic patient characteristics.

## Methods

This cross-sectional study used deidentified hospital inpatient stays with nonmissing data on patient-level demographic, comorbidity, and treatment characteristics and outcomes derived from discharge records and claims extracted from the Vizient Clinical Data Base (CDB), from more than 800 hospitals in the US. The hospitals in our sample have relatively large capacity and volume, with 231 beds on average and 11 843 inpatient discharges per year relative to the American Hospital Association reported national means: 153 beds and 6199 discharges.^21^ All inpatient stays of adults aged 18 years and older between March 1, 2020, and March 31, 2022, with a primary or secondary diagnosis of COVID-19, where the discharge status was known, were included (eFigure 1 in Supplement 1). COVID-19 stays in March 2020 were identified using the interim coding guidelines published by the Centers for Disease Control and Prevention (CDC) in February 2020 (*International Statistical Classification of Diseases and Related Health Problems, Tenth Revision* [*ICD-10*] code B97.29), and stays from April 2020 through March 2022 were identified using *ICD-10* code U07.1. We present results from all of March 2020, but note that sample sizes were relatively small in those early weeks. Patient-level zip codes, based on their residence, were used to merge weekly COVID-19 case and death rate data obtained from the *New York Times* GitHub repository.^22^ This study followed the Strengthening the Reporting of Observational Studies in Epidemiology (STROBE) reporting guideline for cross-sectional studies^23^ and was approved by The University of Texas Health Houston Institutional Review Board, with waiver of informed consent due to the use of secondary deidentified data.

### Outcome Measure

The primary outcome was direct medical costs to the hospital to produce care calculated by Vizient. This measure allows for better comparison of national medical resource use by accounting for geographic differences in the labor costs of medical professionals and hospital-specific attributes.^24^ Vizient classified individual charges (or amounts billed) as directly related to providing patient care (eg, staffing, equipment, and supplies related to the specific care provided)^25^ and adjusted them using the hospital-specific cost to charge ratio calculated from the Centers for Medicare & Medicaid Services cost report data, multiplied by an area wage index to adjust for geographic differences in labor costs.^26,27^ Using the cost to charge ratio is a standard approach to converting charges to costs using facility-level cost accounting data that hospitals are required to submit to the Centers for Medicare & Medicaid Services.^28,29,30,31,32,33^ In adjusted models, we used the risk-adjusted cost measure that Vizient adjusted at the patient level.^25^ All costs reported are in constant January 2022 US dollars.

### Covariates

The following characteristics of the inpatient admission were included in adjusted models to adjust for patient acuity, intensity of treatment, and social determinants of health through proxy measures of sociodemographic information available in the data: hour of admission (vector of hourly indicators); day of week of admission (vector of day of week fixed effects); whether the patient presented to the emergency department (indicator); the weekly hospital volume of inpatient COVID-19 admissions in the week of admission (continuous); whether the admission was a transfer from another hospital, skilled nursing facility, or other health care facility (indicator); and discharge status (categorical). Patient-level risk factors included age (continuous), female sex (indicator), race and ethnicity (categorical), health insurance status (categorical), census geographic divisions (categorical), 29 indicators for the individual comorbidities used in the Elixhauser comorbidity index,^34^ whether COVID-19 was the primary vs secondary diagnosis (indicator), and an indicator for whether the patient died.^23^ Vizient used the Agency for Healthcare Research and Quality Elixhauser Comorbidity software to identify comorbidities, which relies exclusively on secondary diagnoses and excludes diagnoses that were not present on admission.^35^ Several proxy measures for treatment intensity were used. These included length of stay in days (continuous) and whether the patient had any of the following events during the stay (not mutually exclusive): extracorporeal membrane oxygenation (ECMO), invasive mechanical ventilation, noninvasive mechanical ventilation, and ICU admission. Race and ethnicity information was obtained from the Vizient CDB for each inpatient stay and coded as reported in Table 1. Race and ethnicity are demographic characteristics for which differences in COVID-19 infection, hospitalization, morbidity, and mortality rates vary^36^ and can therefore be associated with differences in costs of inpatient treatment. The weekly COVID-19 case rates (per 100 000 residents) in the patient’s zip code at the time of admission were included as indicators of potential hospital capacity constraints.

**Table 1.** Full Sample Characteristics^a^

Characteristic	No. (%)
No. of inpatient stays	1 333 404
Age, mean (SD), y	59.2 (17.5)
Biological sex	
Female	640 854 (48.1)
Male	692 550 (51.9)
Race and ethnicity	
Asian	35 909 (2.7)
Hispanic	181 249 (13.6)
Non-Hispanic Black	292 029 (21.9)
Non-Hispanic White	788 727 (59.2)
Other^b^	17 673 (1.3)
Unavailable^c^	17 817 (1.3)
Primary health insurance	
Private/commercial	411 244 (30.8)
Medicaid	244 479 (18.3)
Medicare	613 266 (46.0)
Military	16 675 (1.3)
Self-pay	43 262 (3.2)
Workers compensation/automobile	4478 (0.3)
Unknown	47 104 (3.5)
Census division of residence	
New England	75 285 (5.7)
Mid-Atlantic	174 935 (13.1)
Midwest	66 709 (5.0)
West North Central	106 183 (8.0)
South Atlantic	271 426 (20.4)
East North Central	250 307 (18.8)
East South Central	52 645 (4.0)
West South Central	157 757 (11.8)
Mountain	96 143 (7.2)
Pacific	82 014 (6.2)
Patient presented to ED	1 079 645 (81.0)
Patient transferred in from another hospital	192 844 (14.5)
COVID-19 principal diagnosis	769 114 (57.7)
Length of stay, mean (SD), d	8.90 (12.2)
ICU stay	360 114 (27.0)
ICU length of stay (includes 0s), mean (SD), d	2.2 (7.3)
ICU length of stay (conditional on ICU stay), mean (SD), d	8.2 (12.3)
Noninvasive mechanical ventilation	105 537 (7.9)
Invasive mechanical ventilation	168 591 (12.6)
Extracorporeal membrane oxygenation	5965 (0.4)
Elixhauser comorbidity indicators	
Alcohol use disorder	103 419 (7.8)
Blood loss/anemia	98 678 (7.4)
Chronic peptic ulcer disease	92 657 (6.9)
Chronic pulmonary disease	85 969 (6.4)
Coagulation deficiency	83 226 (6.2)
Congestive heart failure	87 683 (6.6)
Deficiency anemia	97 528 (7.3)
Depression	108 561 (8.1)
Diabetes with complications	115 131 (8.6)
Diabetes without complications	114 610 (8.6)
Substance use disorder	109 796 (8.2)
Fluid and electrolyte disorders	104 534 (7.8)
HIV/AIDS	98 738 (7.4)
Hypertension	95 311 (7.1)
Hypothyroidism	90 544 (6.8)
Kidney failure	122 250 (9.2)
Liver disease	87 176 (6.5)
Lymphoma	86 605 (6.5)
Metastatic cancer	81 580 (6.1)
Obesity	81 564 (6.1)
Other neurologic disorders	86 177 (6.5)
Paralysis	89 959 (6.7)
Peripheral vascular disorders	99 489 (7.5)
Psychoses	105 792 (7.9)
Pulmonary circulation disorders	113 139 (8.5)
Rheumatoid arthritis/collagen vascular disease	136 920 (10.3)
Solid tumor without metastasis	156 763 (11.8)
Valvular disease	168 127 (12.6)
Weight loss	173 079 (13.0)
Discharge status	
Home	727 057 (54.5)
Hospital	34 409 (2.6)
SNF/rehabilitation facility	59 183 (4.4)
Home health care	177 096 (13.3)
Hospice	21 106 (1.6)
Death	177 582 (13.3)
Unknown	136 971 (10.3)

Abbreviations: ED, emergency department; ICU, intensive care unit; SNF, skilled nursing facility.

^a^ Data from the Vizient Clinical Data Base used by permission of Vizient Inc. All rights reserved.

^b^ Other indicates multiracial.

^c^ Unavailable indicates that information on race and ethnicity was missing from the data.

### Statistical Analysis

Standard descriptive statistics of all measures were calculated. To examine the adjusted mean hospital cost for COVID-19 inpatient treatment, a generalized linear model with a log link function assuming a γ distribution was estimated, adjusting for admission per patient-, treatment-, hospital-, and zip code–level factors described herein. Models were estimated with week fixed effects with SEs clustered at the hospital level. Postestimation adjusted mean costs and the 95% CIs were generated holding the covariates at their means using the margins command in Stata, version 17 (StataCorp LLC). All statistical tests were 2-tailed with a significance level of *P* = .05. Stata and R, version 4.2.1 (R Project for Statistical Computing) were used for analyses.

## Results

The final analytic sample included 1 333 404 inpatient stays with nonmissing costs across 234 teaching and 607 community hospitals from March 2020 through March 2022. The CDC reported 4.58 million hospital admissions from August 2020 through March 2022^7^; our sample included 1 184 293 inpatient stays over the same weeks (26%). Mean (SD) patient age was 59.2 (17.5) years, with 640 854 (48%) women and 692 550 (52%) men. A total of 35 909 (3%) of the patients were Asian, 181 249 (14%) Hispanic, 292 029 (22%) non-Hispanic Black, and 788 727 (59%) non-Hispanic White. Full descriptive statistics are reported in Table 1. Eighty-one percent of the patients presented to the emergency department, 13% received invasive mechanical ventilation, 27% had an ICU stay, and 13% died during their hospital stay. The mean (SD) length of stay was 8.90 (12.16) days. For comparison, the CDC reported that, among patients hospitalized with COVID-19, 6.1% to 13.8% received invasive mechanical ventilation, 18% to 22% had an ICU stay, and mortality rates ranged between 6.5% and 12.6% over the same period.^7^

Across all weeks, the unadjusted mean direct hospital cost per inpatient stay was $13 023 (95% CI, $12 977-$13 070). The mean direct hospital cost per inpatient stay adjusting for patient risk factors was $11 275 (95% CI, $11 252-$11 297); this difference reflects the importance of adjusting for outlier factors in the hospital cost to produce care.

In Table 2, we report how the adjusted mean direct hospital cost varied by key patient demographic characteristics and intensity of treatment (full set of coefficients in eTable 1 in Supplement 1). On average, hospital costs were higher for men vs women: $12 097 (95% CI, $11 994-$12 200) vs $10 654 (95% CI, $10 563-$10 745). There were statistically significant differences across some racial and ethnic categories, although the magnitude was relatively small. There were significant differences by discharge status, with those who went to a skilled nursing facility or rehabilitation facility having the highest adjusted mean hospital costs at $14 005 (95% CI, $13 707-$14 302); the lowest was observed among those who died at $9580 (95% CI, $9472-$9688) or were discharged to hospice at $10 080 (95% CI, $9962-$10 199). We also observed significant differences across geographic regions, with the highest cost in the Pacific division at $12 021 (95% CI, $11 704-$12 338) and the lowest in the New England division at $10 746 (95% CI, $10 606-$10 885). Hospital costs varied by payer, with private insurance at $11 274 (95% CI, $11 251-$11 297), US military insurance at $11 488 (95% CI, $11 393-$11 583), and worker’s compensation plans at $13 106 (95% CI, $12 897-$13 316) having the highest adjusted mean costs.

**Table 2.** Adjusted Mean Costs by Key Patient- and Discharge-Level Characteristics^a^

Variable	Mean cost (95% CI), $^b^
Overall unadjusted mean, 2022 $	13 023 (12 977-13 070)
Overall adjusted mean, 2022 $	11 432 (11 336-11 528)
Patient characteristics	
By biological sex	
Female	10 654 (10 563-10 745)
Male	12 097 (11 994-12 200)
By race and ethnicity	
Asian	11 724 (11 562-11 886)
Black	11 370 (11 258-11 481)
Hispanic	11 479 (11 371-11 586)
Other^c^	11 700 (11 484-11 916)
White	11 417 (11 313-11 522)
Missing	11 702 (11 467-11 937)
By health insurance coverage	
Private/commercial	11 274 (11 251-11 297)
Medicaid	10 629 (10 602-10 656)
Medicare	11 609 (11 589-11 629)
Military	11 488 (11 393-11 583)
Self-pay	11 085 (11 025-11 145)
Workers compensation/automobile	13 106 (12 897-13 316)
Unknown	11 274 (11 251-11 297)
By census division of residence	
New England	10 746 (10 606-10 885)
Mid-Atlantic	11 154 (10 951-11 358)
Midwest	11 625 (11 252-11 999)
West North Central	11 348 (11 047-11 648)
South Atlantic	11 324 (11 154-11 495)
East South Central	11 761 (11 455-12 066)
West South Central	11 898 (11 605-12 191)
Mountain	11 694 (11 454-11 934)
Pacific	12 021 (11 704-12 338)
By discharge status	
Home	10 812 (10 716-10 909)
Hospital	10 494 (10 363-10 625)
SNF/rehabilitation facility	14 005 (13 707-14 302)
Home health care	11 620 (11 503-11 737)
Hospice	10 080 (9962-10 199)
Death	9580 (9472-9688)
Treatment intensity	
Presented to ED = yes	11 519 (11 425-11 612)
Presented to ED = no	11 117 (10 915-11 318)
Transferred in = yes	12 520 (12 368-12 671)
Transferred in = no	11 189 (11 088-11 289)
COVID-19 principal diagnosis = yes	11 794 (11 695-11 893)
COVID-19 principal diagnosis = no	10 991 (10 876-11 106)
ICU stay = yes	12 490 (12 306-12 674)
ICU stay = no	10 724 (10 618-10 830)
Noninvasive mechanical ventilation = yes	12 621 (12 488-12 755)
Noninvasive mechanical ventilation = no	11 336 (11 239-11 433)
Invasive mechanical ventilation = yes	20 941 (20 685-21 198)
Invasive mechanical ventilation = no	9614 (9530-9697)
ECMO = yes	36 484 (34 685-38 284)
ECMO = no	11 266 (11 173-11 358)

Abbreviations: ECMO, extracorporeal membrane oxygenation; ED, emergency department; ICU, intensive care unit; SNF, skilled nursing facility.

^a^ Data from the Vizient Clinical Data Base used by permission of Vizient Inc. All rights reserved.

^b^ Means and 95% CIs represent the mean adjusted inpatient costs for each category (except where noted elsewhere), which were obtained postestimation from the generalized linear model adjusted for the hour and day of week of admission; whether the patient presented to the ED; the weekly hospital volume of inpatient COVID-19 admissions in the week of admission; whether the admission was a transfer from another hospital, skilled nursing facility, or other health care facility; patient characteristics (age, female sex, race and ethnicity, health insurance status, census geographic divisions, 29 indicators for the individual comorbidities used in the Elixhauser comorbidity index, and whether COVID-19 was the primary diagnosis); treatment measures (length of stay in days); whether the patient had any of the following events during the stay (not mutually exclusive): ECMO, invasive mechanical ventilation, noninvasive mechanical ventilation, ICU admission, and weekly COVID-19 case rates (per 100 000 residents) in the patient’s zip code at the time of admission; and week fixed effects. Table 1 provides a full list of sample characteristics, and eTable 1 in Supplement 1 reports the full set of generalized linear model coefficients.

^c^ Other indicates multiracial.

In addition, we show the adjusted mean costs by characteristics of treatment (noting that these are not mutually exclusive). The most significant factor explaining differences in the hospital cost of care was whether the patient had ECMO during the stay, which resulted in an adjusted mean hospital cost of $36 484 (95% CI, $34 685-$38 284) compared with those without ECMO: $11 266 (95% CI, $11 173-$11 358). Those using invasive mechanical ventilation had an adjusted mean cost of $20 941 (95% CI, $20 685-$21 198) vs $9614 (95% CI, $9530-$9697) for those without this treatment. Given this significant factor in cost differences, we also examined the cost differences over time among patients with and without ECMO or any mechanical ventilation (eFigure 2 in Supplement 1); the hospital cost difference widened during the Omicron wave. The fraction of stays that included ECMO or mechanical ventilation over time started at 23% of stays at the end of March 2020 (ignoring early weeks, which had few inpatient stays), peaking at 30% in late 2021, and then decreasing to less than 20% by the end of March 2022.

**Figure 1.**
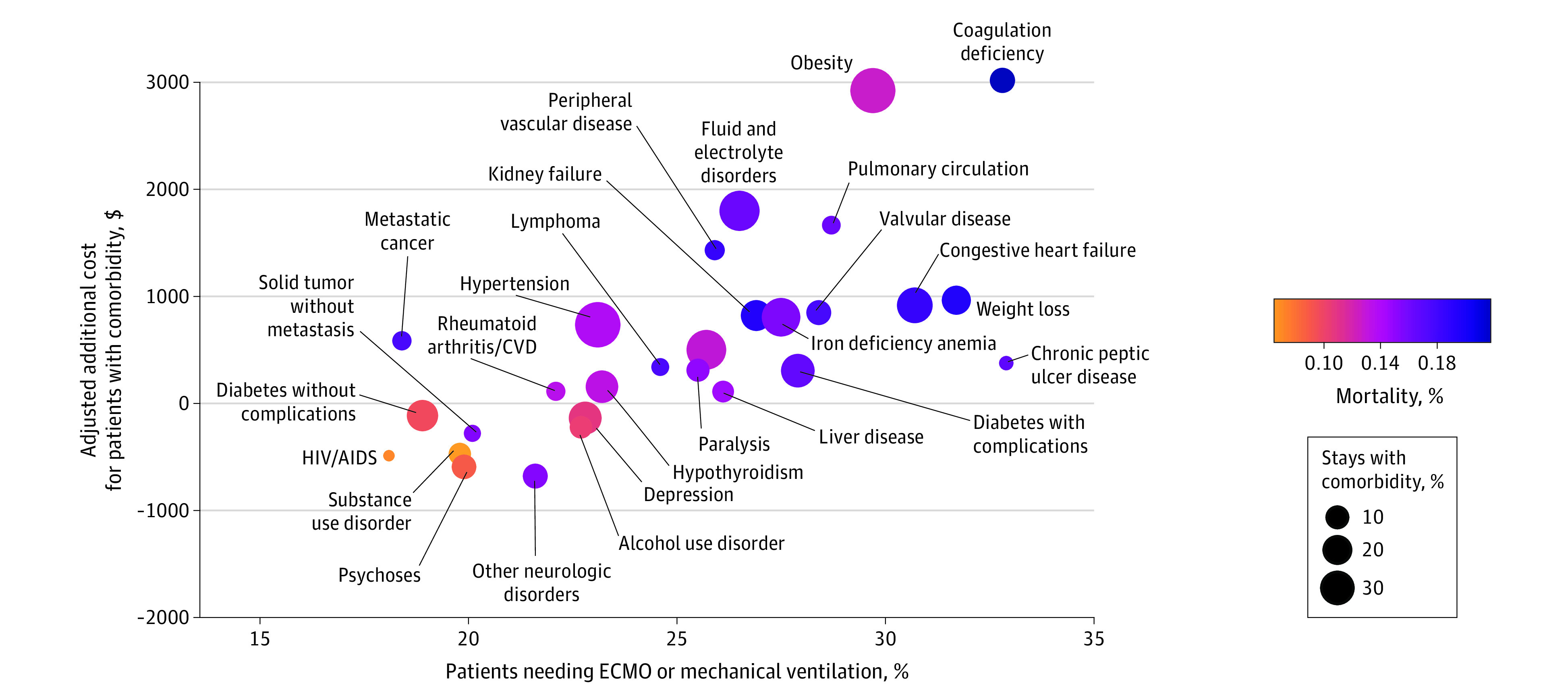
Mean Additional Inpatient Costs, Percentage Needing Extracorporeal Membrane Oxygenation (ECMO) or Mechanical Ventilation, and Mortality Percentage by Comorbidity The points show the difference in the mean adjusted inpatient costs for patients with and without each comorbidity derived from the generalized linear model adjusted for the same covariates as listed in footnote *b* of Table 2. The size of the point reflects the percentage of the sample with a given comorbidity (eg, 32% of stays included an obesity diagnosis). The shading of the point reflects the mortality rate of patients with a given comorbidity (eg, 14% of patients with an obesity diagnosis died). Only comorbidities with a statistically significant difference in costs are shown (see full list in eTable 2 in Supplement 1). CVD indicates collagen vascular disease. Data obtained from the Vizient Clinical Data Base, used by permission of Vizient Inc. All rights reserved.

In Figure 1 we show how the presence of one of the Elixhauser comorbidities was associated with differences in adjusted hospital costs when statistically significant. Inpatients with the highest hospital costs were more likely to have needed ECMO or mechanical ventilation but did not necessarily have the highest mortality rates. For example, those with obesity had $2924 in additional treatment costs compared with those without obesity ($13 280 vs $10 356) (eTable 2 in Supplement 1). Thirty percent of patients with obesity (95% CI, 2%-30%) required ECMO or mechanical ventilation, which was significantly higher than among all patients (21%; 95% CI, 21%-21%). Mortality rates among those with obesity were similar to the overall sample. Patients with coagulation deficiency had similarly high rates of ECMO or any mechanical ventilation and $3017 in additional inpatient costs. However, 22% of these patients died (95% CI, 22%-22%). The size of the points in Figure 1 reflects the relative percentage of the sample with the comorbidity. For example, 32% of the patients had an obesity diagnosis, whereas only 5% had a coagulation deficiency diagnosis (eTable 2 and eTable 3 in Supplement 1). Increases in hospital costs paralleled increases in the use of ECMO or mechanical ventilation overall (eFigure 2 in Supplement 1); in addition, costs increased by 35% among those needing ECMO or mechanical ventilation (eFigure 2 in Supplement 1).

**Figure 2.**
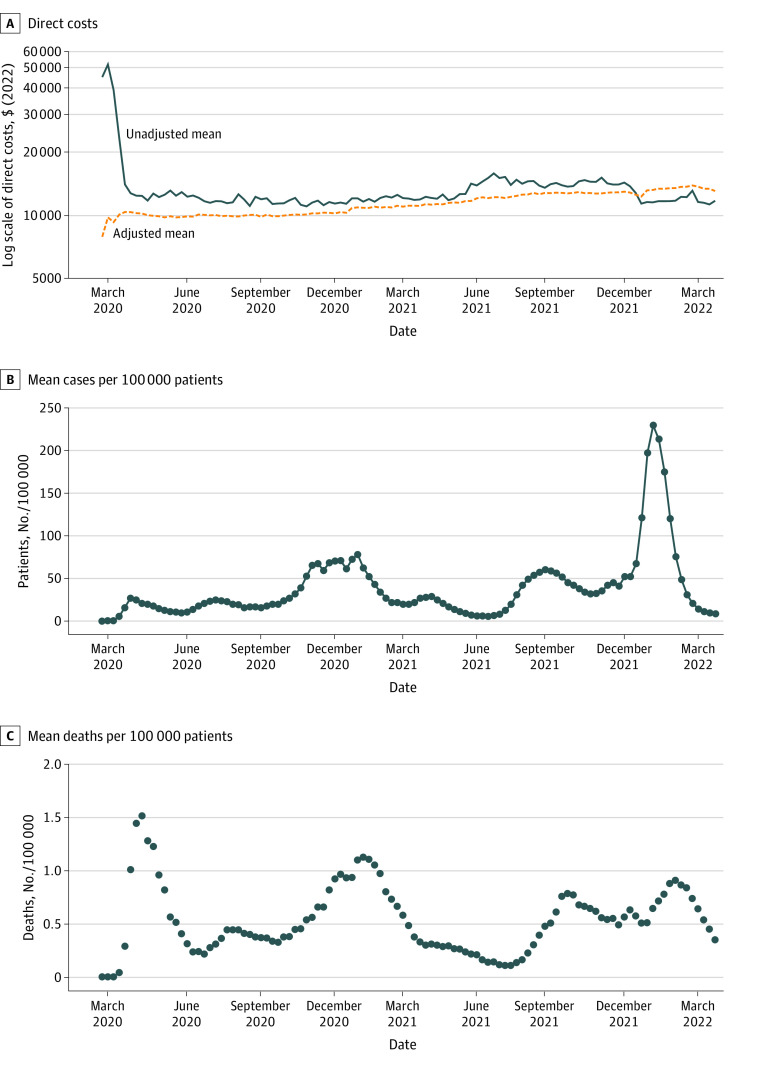
Mean COVID-19 Inpatient Direct Costs, Unadjusted and Adjusted, and Case and Death Rates Over Time A, Direct costs of hospitalization. B, Mean number of COVID-19 cases per 100 000 in the patient's county of residence. C, Mean number of deaths due to COVID-19 per 100 000 in the patient's county of residence. The adjusted cost estimates were derived postestimation from the generalized linear model, including the same covariates as listed in footnote *b* of Table 2. Data obtained from the Vizient Clinical Data Base, used by permission of Vizient Inc. All rights reserved.

Figure 2A shows how the unadjusted mean inpatient costs per stay changed over time. Unadjusted hospital costs were considerably higher than average and more varied during the early weeks of the pandemic. Adjusted mean hospital costs were $10 394 (95% CI, $10 228-$10 559) per stay at the end of March 2020, increasing to $13 072 (95% CI, $12 528-$13 617) by the end of March 2022. This is a 26% increase over a 2-year period, which is significantly more than the average percentage change in medical care costs in the average US city of 4.7% over the same time. Although there was variation over time, significant changes in hospital costs did not coincide with the general trends in case or death rates (Figure 2B and C).

## Discussion

This study contributes to the evidence on the mean hospital cost of inpatient care for patients with COVID-19 in several ways. First, we used the Vizient CDB, a database covering 97% of the academic centers across the US with more than 10 million inpatient admissions per year.^37^ Our sample included more than 1.3 million stays from March 1, 2020, through March 31, 2022. Second, we controlled for actual case rates in a patient’s zip code to better account for increased demand. Third, we examined differences in treatment costs by key patient characteristics, hospital characteristics, and treatment intensity. Fourth, we used a measure of the direct cost to produce care as opposed to the price or the charges patients and payers face, which allows for comparison across facilities and improves on using charges or payments that do not account for premiums, deductibles, and taxes devoted toward health care expenditures.^1,5,38^

Capturing nearly all academic medical centers across the US, our study of 1.3 million stays from March 2020 through March 2022 found an inpatient mean cost to provide care at $11 275 (in 2022 $), which suggests an approximate direct medical resource use or hospital cost to deliver these services of $15.03 billion. Extrapolating this to the total 6.2 million hospital admissions the CDC reported from August 2020 through July 15, 2023,^7^ would suggest an aggregate direct cost for providing inpatient care to treat patients with COVID-19 in the US at $70 billion, not including health care costs for outpatient treatment, testing, immunizations, or emergency department visits that did not result in an admission. This estimate excludes the costs associated with lost days of work, lost years of productive life, and added financial burdens to families.

We found that some chronic conditions resulted in significantly higher hospital costs, and it tended to be the case that those patients were more likely to use ECMO or mechanical ventilation. This might suggest a higher severity of COVID-19 disease among these patients, but these higher-cost comorbidities were not consistently associated with longer lengths of stay, greater use of ICU, more comorbidities, or higher mortality rates (full results in eTable 2 and eTable 3 in Supplement 1).

The adjusted mean cost for inpatient treatment of COVID-19 increased significantly: 26% over a 2-year period compared with a 2% to 5% average annual medical cost inflation.^39^ In general, the change in hospital costs over time was not associated with the changes in case and mortality rates. ECMO or mechanical ventilation use increased over time until the end of 2021 (eFigure 2 in Supplement 1). Among patients treated with ECMO or mechanical ventilation, costs increased even more, by 35%.

The composition of patients admitted for treatment may very well have changed over time in ways that could affect changes in hospital costs. However, the direction of this influence is unclear. For example, if patients with the greatest risks were more likely to obtain vaccines, we might expect costs to decrease over time. However, if patients with fewer comorbidities were more likely to obtain vaccines, we might expect costs to increase over time.^40^

In addition, there may be factors on the supply side involved. The American Hospital Association noted 3 key inflationary pressures increasing the hospital cost of delivering inpatient care since the onset of the pandemic—the costs of drugs, labor, and supplies—which are estimated in our data in total for each stay, but we cannot examine these components separately in our data.^41^ Hospitals’ cost of prescription drug treatments used to treat COVID-19 has increased over time as emergency use authorizations and US Food and Drug Administration approvals were granted, manufacturers’ donated doses were depleted, and the federal government’s program of purchasing and distributing therapeutics waned.^42^ The American Hospital Association report also noted that for the Delta and Omicron surges, the US government was no longer providing financial assistance to hospitals through the COVID-19 Provider Relief Fund. The loss of federal assistance may be associated with the increased need for reimbursable charges, which would be reflected in the Vizient data.

### Strengths and Limitations

This study has key strengths, including a large sample size using data from a large group purchasing organization that is similar to the Premier Healthcare Database (the data source used in previous studies^9,10,11^), a national sampling frame with hospitals of varying size and capability and an extended observation period. Limitations of the study include measurement error from the use of administrative data.^43,44,45^ Measurement error may bias our estimates downward due to, for example, missed comorbidities or deaths that happen after discharge. The costs measure was derived from hospital charges and adjusted using standard approaches but may still be affected by measurement error and does not reflect patient or payer costs. The hospitals in this sample may not be representative of all hospitals or all COVID-19 inpatient stays but are representative of academic medical centers, which typically have more critically ill patients.^46^

The costs presented herein are aggregated over individual hospital admissions. Some patients likely had recurrent admissions; thus, the total hospitalization cost per patient might be greater than that reported herein for single admissions. Shrestha et al^11^ reported that 94.9% of the inpatients in the Premier Healthcare database had only 1 hospitalization from March 2020 through July 2021.

## Conclusions

In this cross-sectional study of more than 1.3 million inpatient admissions for treatment of COVID-19 from March 2020 through March 2022, we estimated an average national medical resource use or hospital cost to deliver care per COVID-19 inpatient stay at $11 275. Hospital costs increased more than 5 times the rate of medical inflation over this period. This was explained partly by changes in the use of ECMO, which also increased over time. Nonetheless, costs to provide inpatient care increased even as care practices changed, vaccination rates increased, and the variants of concern evolved.

## Notes

Supplement 1. **eFigure 1.** Analytic Sample Construction Flow **eTable 1.** GLM Regression Coefficients **eTable 2.** Average Adjusted Costs, Length of Stay (LOS) by Presence of Elixhauser Comorbidity **eTable 3.** Average Adjusted Mortality Rate, Age, and ECMO/Mechanical Ventilation Use, by Presence of Elixhauser Comorbidity **eFigure 2.** Adjusted Costs of Inpatient by ECMO/Mechanical Ventilation and Week

Supplement 2.
Data Sharing Statement
