# Supplementary material for: Inpatient Costs of Treating Patients With COVID-19
Source: JAMA Netw Open. 2024 Jan 3;7(1):e2350145. doi: 10.1001/jamanetworkopen.2023.50145 (PMC10765267; doi:10.1001/jamanetworkopen.2023.50145)
Supplement: Supplement 1. — eFigure 1. Analytic Sample Construction Flow eTable 1. GLM Regression Coefficients eTable 2. Average Adjusted Costs, Length of Stay (LOS) by Presence of Elixhauser Comorbidity eTable 3. Average Adjusted Mortality Rate, Age, and ECMO/Mechanical Ventilation Use, by Presence of Elixhauser Comorbidity eFigure 2. Adjusted Costs of Inpatient by ECMO/Mechanical Ventilation and Week [file jamanetwopen-e2350145-s001.pdf]

Supplementary Online Content

Kapinos KA, Peters RM Jr, Murphy RE, Hohmann SF, Podichetty A, Greenberg RS. Inpatient costs of treating patients with COVID-19. *JAMA Netw Open*. 2024;7(1):e2350145. doi:10.1001/jamanetworkopen.2023.50145

eFigure 1. Analytic Sample Construction Flow

eTable 1. GLM Regression Coefficients

eTable 2. Average Adjusted Costs, Length of Stay (LOS) by Presence of Elixhauser Comorbidity

eTable 3. Average Adjusted Mortality Rate, Age, and ECMO/Mechanical Ventilation Use, by Presence of Elixhauser Comorbidity

eFigure 2. Adjusted Costs of Inpatient by ECMO/Mechanical Ventilation and Week

This supplementary material has been provided by the authors to give readers additional information about their work.

© 2024 Kapinos KA et al. *JAMA Network Open.*

eFigure 1. Analytic Sample Construction Flow Diagram

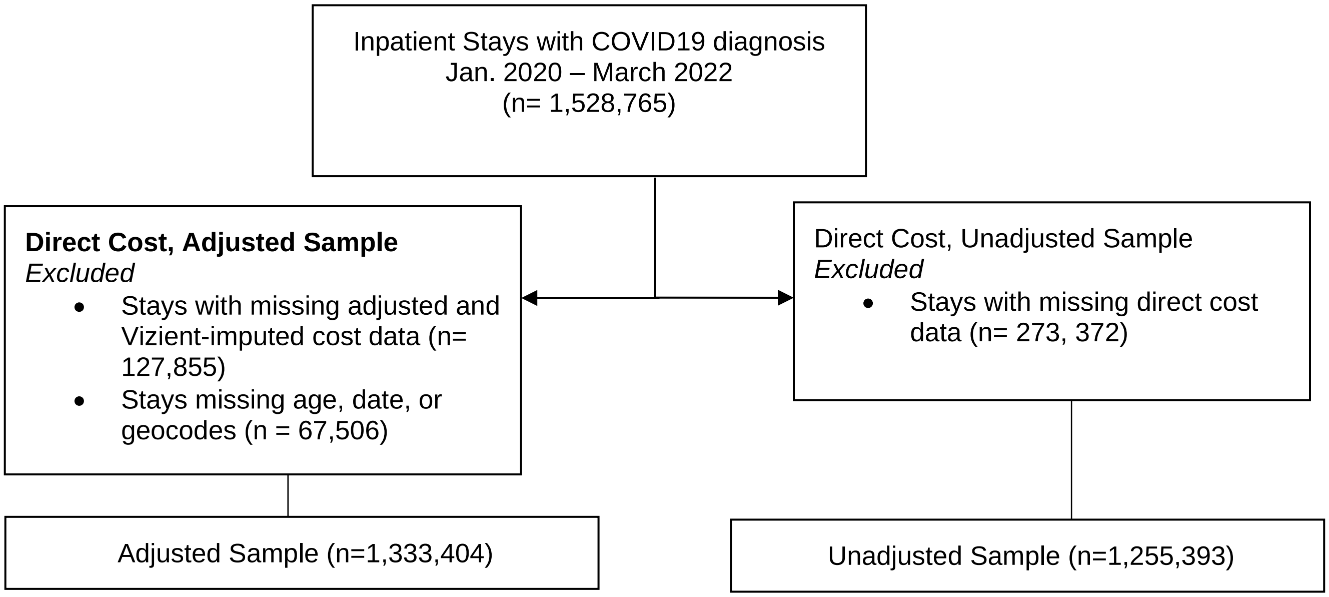

© 2024 Kapinos KA et al. *JAMA Network Open.*

eTable 1. GLM Regression Coefficients

	Coefficient	95% CI
Patient Started in ED	0.036	(0.018,0.053)
ICU Stay	0.152	[0.19,0.22]
Length of Stay (Days)	0.010	(0.009,0.01)
Age	0.003	(0.003,0.003)
Hour of admission (24th is ref)		
1	-0.003	(-0.016,0.009)
2	-0.010	(-0.023,0.004)
3	-0.011	(-0.023,0.002)
4	-0.023	(-0.036,-0.01)
5	0.000	(-0.015,0.014)
6	-0.023	(-0.037,-0.009)
7	-0.035	(-0.048,-0.021)
8	-0.026	(-0.04,-0.013)
9	-0.026	(-0.039,-0.013)
10	-0.026	(-0.038,-0.013)
11	-0.022	(-0.035,-0.01)
12	-0.021	(-0.033,-0.008)
13	-0.021	(-0.032,-0.009)
14	-0.025	(-0.036,-0.013)
15	-0.026	(-0.037,-0.014)
16	-0.022	(-0.034,-0.01)
17	-0.020	(-0.032,-0.008)
18	-0.020	(-0.032,-0.009)
19	-0.018	(-0.029,-0.007)
20	-0.023	(-0.035,-0.011)
21	-0.020	(-0.031,-0.008)
22	-0.020	(-0.032,-0.008)
23	-0.016	(-0.028,-0.004)
99	-0.044	(-0.082,-0.007)
Sex (unknown is ref)		
Male	-0.053	(-0.187,0.08)
Female	-0.180	(-0.314,-0.046)
Race/Ethnicity (Missing is ref)		
White	-0.039	(-0.063,-0.014)
Black	-0.043	(-0.068,-0.018)
Asian	-0.012	(-0.038,0.014)
Hispanic	-0.033	(-0.057,-0.009)
Other	-0.014	(-0.043,0.015)
Unknown	-0.014	(-0.044,0.016)

© 2024 Kapinos KA et al. *JAMA Network Open.*

Admission Day of Week (Sunday is ref)		
Monday	0.000	(-0.004,0.004)
Tuesday	-0.004	(-0.008,0)
Wednesday	-0.005	(-0.009,-0.001)
Thursday	-0.004	(-0.008,0)
Friday	-0.003	(-0.006,0.001)
Saturday	0.000	(-0.004,0.003)
Number of Days in ICU	-0.002	(-0.003,-0.001)
Health Insurance (Private/Commercial is ref)		
Medicaid	0.037	(0.03,0.043)
Medicare	0.047	(0.031,0.062)
Military	0.050	(0.037,0.064)
Self-pay	0.220	(0.191,0.249)
Workers Compensation/Auto	0.056	(0.049,0.064)
Unknown	0.000	0
Extracorporeal membrane oxygenation	1.175	(1.125,1.225)
Invasive Mechanical Ventilation	0.779	(0.764,0.793)
Non-Invasive Mechanical Ventilation	0.107	(0.099,0.116)
Number of COVID-19 stays at hospital on day of admission	0.000	(0,0)
Census Division (East North Central is ref)		
Middle Atlantic	-0.013	(-0.034,0.008)
New England	-0.050	(-0.067,-0.034)
West North Central	0.004	(-0.024,0.032)
South Atlantic	0.002	(-0.016,0.02)
East South Central	0.040	(0.012,0.068)
West South Central	0.051	(0.026,0.077)
Mountain	0.034	(0.011,0.057)
Pacific	0.062	(0.033,0.091)
Teaching hospital	0.037	(0.023,0.051)
COVID-19 Principal Diagnosis	0.071	(0.062,0.079)
Patient Transferred in from Another Hospital	0.112	(0.1,0.125)
Number of weeks since Jan 1. 2020		
6	0.251	(-0.456,0.958)
7	1.766	(0.796,2.735)
9	0.944	(0.211,1.677)

© 2024 Kapinos KA et al. *JAMA Network Open.*

10	1.679	(1.41,1.947)
11	1.889	(1.692,2.086)
12	1.837	(1.69,1.984)
13	1.920	(1.779,2.06)
14	1.948	(1.808,2.088)
15	1.947	(1.807,2.087)
16	1.935	(1.794,2.076)
17	1.931	(1.791,2.072)
18	1.913	(1.772,2.054)
19	1.906	(1.766,2.046)
20	1.901	(1.76,2.042)
21	1.889	(1.748,2.031)
22	1.902	(1.761,2.043)
23	1.890	(1.744,2.035)
24	1.893	(1.752,2.033)
25	1.900	(1.76,2.041)
26	1.898	(1.758,2.038)
27	1.921	(1.78,2.062)
28	1.917	(1.778,2.056)
29	1.911	(1.771,2.051)
30	1.915	(1.775,2.056)
31	1.902	(1.763,2.042)
32	1.908	(1.769,2.047)
33	1.901	(1.762,2.04)
34	1.899	(1.759,2.04)
35	1.909	(1.77,2.049)
36	1.916	(1.774,2.057)
37	1.919	(1.779,2.058)
38	1.894	(1.755,2.033)
39	1.919	(1.779,2.06)
40	1.902	(1.76,2.044)
41	1.902	(1.762,2.042)
42	1.909	(1.768,2.05)
43	1.916	(1.775,2.057)
44	1.921	(1.78,2.061)
45	1.918	(1.778,2.057)
46	1.921	(1.782,2.06)
47	1.934	(1.794,2.073)
48	1.933	(1.793,2.072)
49	1.941	(1.8,2.081)
50	1.939	(1.8,2.079)
51	1.932	(1.792,2.072)
52	1.947	(1.807,2.087)

© 2024 Kapinos KA et al. *JAMA Network Open.*

53	1.939	(1.8,2.079)
54	1.991	(1.851,2.13)
55	1.998	(1.858,2.138)
56	1.994	(1.854,2.133)
57	1.992	(1.852,2.132)
58	2.006	(1.865,2.147)
59	1.995	(1.855,2.135)
60	2.002	(1.862,2.142)
61	1.997	(1.857,2.137)
62	2.016	(1.875,2.157)
63	2.006	(1.865,2.147)
64	2.017	(1.877,2.156)
65	2.016	(1.876,2.157)
66	2.014	(1.874,2.155)
67	2.034	(1.895,2.172)
68	2.027	(1.887,2.168)
69	2.034	(1.894,2.174)
70	2.031	(1.891,2.171)
71	2.046	(1.907,2.186)
72	2.052	(1.91,2.194)
73	2.048	(1.907,2.189)
74	2.067	(1.926,2.207)
75	2.068	(1.929,2.207)
76	2.097	(1.955,2.238)
77	2.108	(1.965,2.251)
78	2.098	(1.957,2.24)
79	2.109	(1.967,2.252)
80	2.109	(1.97,2.247)
81	2.100	(1.962,2.238)
82	2.112	(1.974,2.251)
83	2.123	(1.984,2.261)
84	2.137	(1.999,2.274)
85	2.138	(1.999,2.277)
86	2.156	(2.016,2.295)
87	2.140	(2.001,2.28)
88	2.155	(2.016,2.294)
89	2.153	(2.014,2.292)
90	2.158	(2.018,2.298)
91	2.156	(2.015,2.296)
92	2.150	(2.01,2.29)
93	2.156	(2.016,2.296)
94	2.165	(2.024,2.306)
95	2.153	(2.012,2.293)

© 2024 Kapinos KA et al. *JAMA Network Open.*

96	2.154	(2.013,2.295)
97	2.149	(2.007,2.291)
98	2.150	(2.01,2.29)
99	2.158	(2.017,2.299)
100	2.161	(2.021,2.301)
101	2.161	(2.021,2.302)
102	2.169	(2.029,2.309)
103	2.159	(2.019,2.299)
104	2.136	(1.996,2.276)
105	2.116	(1.976,2.256)
106	2.183	(2.043,2.322)
107	2.187	(2.047,2.327)
108	2.201	(2.06,2.342)
109	2.200	(2.059,2.341)
110	2.207	(2.065,2.348)
111	2.208	(2.066,2.35)
112	2.222	(2.079,2.364)
113	2.223	(2.079,2.367)
114	2.237	(2.091,2.383)
115	2.223	(2.078,2.368)
116	2.201	(2.058,2.344)
117	2.201	(2.055,2.346)
Elixhauser comorbidity indicators:		
Alcohol Use Disorder	-0.018	(-0.027,-0.009)
Blood Loss/ Anemia	-0.159	(-0.177,-0.14)
Chronic Peptic Ulcer Disease	0.030	(0.009,0.051)
Chronic Pulmonary Disease	0.042	(0.039,0.045)
Coagulation Deficiency	0.253	(0.244,0.262)
Congestive Heart Failure	0.074	(0.07,0.079)
Deficiency Anemia	0.077	(0.072,0.081)
Depression	-0.007	(-0.011,-0.004)
Diabetes with complications	0.022	(0.018,0.026)
Diabetes without complications	-0.019	(-0.022,-0.015)
Substance Use Disorder	-0.032	(-0.041,-0.024)
Fluid Electro Disorders	0.157	(0.152,0.162)
HIV/AIDS	-0.057	(-0.111,-0.003)
Hypertension	0.042	(0.036,0.048)
Hypothyroidism	0.010	(0.006,0.013)
Liver Disease	0.014	(0.007,0.02)
Lymphoma	0.037	(0.024,0.05)
Metastatic Cancer	0.060	(0.049,0.07)

© 2024 Kapinos KA et al. *JAMA Network Open.*

Obesity	0.266	(0.262,0.27)
Other Neurological Disorders	-0.065	(-0.07,-0.06)
Paralysis	0.039	(0.031,0.046)
Peripheral Vascular Disorders	0.124	(0.113,0.134)
Psychoses	-0.041	(-0.048,-0.035)
Pulmonary Circulation Disorders	0.139	(0.13,0.149)
Renal Failure	0.067	(0.062,0.072)
Rheumatoid Arthritis/Collagen Vascular Disease	0.011	(0.003,0.019)
Solid Tumor without Metastasis	-0.035	(-0.045,-0.025)
Valvular Disease	0.072	(0.064,0.079)
Weight Loss	0.098	(0.089,0.106)
Died	-0.520	(-0.532,-0.509)
Discharge Status (unknown is ref)		
Home	-0.399	(-0.409,-0.389)
Hospital	-0.429	(-0.443,-0.414)
SNF/Rehab	-0.140	(-0.162,-0.118)
Home Health	-0.327	(-0.337,-0.317)
Hospice	-0.469	(-0.482,-0.456)
Average Cases per 100k in zip code	0.000	(0,0)
Constant	6.893	(6.701,7.085)
N	1333404	

Notes: Data from the Vizient Clinical Data Base used by permission of Vizient, Inc. All rights reserved. Standard errors clustered at the hospital level.
95% confidence intervals in parenthesis

eTable 2. Average Adjusted Costs, Length of Stay (LOS) by Presence of Elixhauser Comorbidity

	Mean Additional Cost	# of Stays	% of Sample	Length of Stay (Days)		Included ICU Stay	
				Mean	95% CI	Mean	95% CI
**Coagulation Deficiency**	$3,017	71316	5%	11.83	(11.73,11.93)	40%	(39.5,40.2)
**Obesity**	$2,924	420057	32%	10.35	(10.31,10.4)	32%	(32,32.3)
**Fluid Electro Disorders**	$1,800	307509	23%	10.21	(10.17,10.25)	34%	(33.6,34)
**Pulmonary Circulation Disorders**	$1,667	20177	2%	10.50	(10.32,10.67)	37%	(36.4,37.7)
**Peripheral Vascular Disorders**	$1,431	28500	2%	11.35	(11.2,11.51)	35%	(34,35.1)
**Weight Loss**	$966	116364	9%	15.20	(15.1,15.31)	39%	(38.5,39)
**Congestive Heart Failure**	$918	218789	16%	10.92	(10.86,10.98)	35%	(35.1,35.5)
**Valvular Disease**	$848	67711	5%	11.68	(11.57,11.79)	35%	(34.3,35)
**Renal Failure**	$822	141012	11%	10.80	(10.73,10.86)	34%	(33.3,33.7)
**Deficiency Anemia**	$805	274142	21%	11.91	(11.85,11.97)	34%	(33.7,34)
**Hypertension**	$735	427519	32%	9.584	(9.55,9.618)	29%	(29.2,29.5)
**Metastatic Cancer**	$585	23469	2%	10.17	(10.01,10.33)	26%	(25.2,26.3)
**Chronic Pulmonary Disease**	$503	297397	22%	9.620	(9.574,9.666)	29%	(28.6,29)
**Chronic Peptic Ulcer Disease**	$378	3921	0%	15.54	(15.01,16.07)	43%	(41.7,44.8)
**Lymphoma**	$341	16010	1%	10.86	(10.66,11.07)	28%	(27.7,29.1)
**Paralysis**	$312	51598	4%	12.57	(12.43,12.71)	36%	(35.8,36.6)
**Diabetes with complications**	$306	183224	14%	10.95	(10.89,11.01)	34%	(33.9,34.3)
**Hypothyroidism**	$157	166092	12%	9.726	(9.665,9.788)	28%	(28.1,28.5)
**Rheumatoid Arthritis/Collagen Vascular Disease**	$116	22707	2%	9.446	(9.302,9.591)	29%	(28.2,29.4)
**Liver Disease**	$112	39868	3%	11.15	(11.01,11.29)	34%	(34,34.9)
**Diabetes without complications**	-$114	147560	11%	7.557	(7.509,7.606)	25%	(25.2,25.6)
**Depression**	-$137	170541	13%	10.63	(10.56,10.71)	28%	(27.6,28)
**Alcohol Use Disorder**	-$223	46292	3%	11.05	(10.9,11.21)	34%	(33.1,33.9)
**Solid Tumor without Metastasis**	-$279	12424	1%	9.898	(9.704,10.09)	28%	(27.3,28.8)
**Substance Use Disorder**	-$472	44845	3%	10.97	(10.81,11.14)	30%	(29.2,30)
**HIV/AIDS**	-$488	646	0%	13.53	(12,15.07)	31%	(26.9,34.1)
**Psychoses**	-$593	64876	5%	10.75	(10.62,10.87)	27%	(26.3,27)
**Other Neurological Disorders**	-$679	68266	5%	10.30	(10.21,10.39)	29%	(28.5,29.2)
**Blood Loss/ Anemia**	-$1,669	9081	1%	7.586	(7.345,7.828)	24%	(22.9,24.7)

© 2024 Kapinos KA et al. *JAMA Network Open.*

Notes: Data from the Vizient Clinical Data Base used by permission of Vizient, Inc. All rights reserved. Derived using margins command post-estimation of GLM (see Supplemental Table 1 for full set of coefficients). 95% CI in parentheses.

eTable 3. Average Adjusted Mortality Rate, Age, and ECMO/Mechanical Ventilation Use, by Presence of Elixhauser Comorbidity

	Death		Age		ECMO or Mechanical Ventilation Use	
	Mean (%)	95% CI	Mean	95% CI	Mean (%)	95% CI
**Coagulation Deficiency**	22%	(21.5,22.1)	63.14	(63.02,63.26)	33%	(32.4,33.1)
**Obesity**	13%	(12.6,12.8)	56.10	(56.05,56.15)	30%	(29.6,29.8)
**Fluid Electro Disorders**	16%	(16.2,16.4)	63.26	(63.2,63.31)	27%	(26.3,26.6)
**Pulmonary Circulation Disorders**	16%	(15.8,16.8)	62.36	(62.15,62.57)	29%	(28.1,29.4)
**Peripheral Vascular Disorders**	19%	(18.1,19)	70.80	(70.65,70.95)	26%	(25.4,26.4)
**Weight Loss**	20%	(19.5,19.9)	66.29	(66.2,66.37)	32%	(31.5,32)
**Congestive Heart Failure**	19%	(18.5,18.8)	68.89	(68.84,68.95)	31%	(30.5,30.9)
**Valvular Disease**	18%	(17.7,18.2)	70.33	(70.22,70.43)	28%	(28.1,28.8)
**Renal Failure**	20%	(19.4,19.8)	68.81	(68.74,68.88)	27%	(26.7,27.2)
**Deficiency Anemia**	16%	(15.6,15.8)	62.49	(62.42,62.55)	28%	(27.3,27.6)
**Hypertension**	14%	(13.9,14.1)	66.11	(66.06,66.15)	23%	(23,23.2)
**Metastatic Cancer**	18%	(17.3,18.3)	65.39	(65.22,65.56)	18%	(17.9,18.9)
**Chronic Pulmonary Disease**	13%	(13,13.3)	63.37	(63.31,63.43)	26%	(25.5,25.8)
**Chronic Peptic Ulcer Disease**	17%	(15.7,18.1)	65.34	(64.89,65.8)	33%	(31.4,34.4)
**Lymphoma**	18%	(17.1,18.3)	66.80	(66.59,67.01)	25%	(23.9,25.2)
**Paralysis**	15%	(14.7,15.4)	64.07	(63.94,64.2)	26%	(25.1,25.8)
**Diabetes with complications**	17%	(16.5,16.8)	65.11	(65.05,65.17)	28%	(27.7,28.1)
**Hypothyroidism**	14%	(13.3,13.6)	67.10	(67.03,67.17)	23%	(23,23.4)
**Rheumatoid Arthritis/Collagen Vascular Disease**	14%	(13.2,14.1)	64.82	(64.62,65.01)	22%	(21.6,22.6)
**Liver Disease**	15%	(14.3,15)	58.23	(58.08,58.37)	26%	(25.7,26.5)
**Diabetes without complications**	10%	(9.8,10.1)	61.89	(61.82,61.97)	19%	(18.7,19.1)
**Depression**	11%	(10.8,11.1)	61.35	(61.27,61.43)	23%	(22.6,23)
**Alcohol Use Disorder**	10%	(10.1,10.6)	54.15	(54.01,54.28)	23%	(22.3,23)
**Solid Tumor without Metastasis**	16%	(14.9,16.2)	69.54	(69.33,69.75)	20%	(19.4,20.8)
**Substance Use Disorder**	7%	(6.2,6.7)	47.21	(47.07,47.35)	20%	(19.5,20.2)
**HIV/AIDS**	7%	(5.1,9.1)	50.44	(49.36,51.52)	18%	(15.1,21.1)
**Psychoses**	9%	(8.8,9.3)	56.26	(56.14,56.39)	20%	(19.6,20.2)
**Other Neurological Disorders**	15%	(15.1,15.6)	67.53	(67.41,67.65)	22%	(21.3,21.9)
**Blood Loss/ Anemia**	7%	(6.2,7.3)	41.46	(41.05,41.88)	14%	(12.8,14.2)

Notes: Data from the Vizient Clinical Data Base used by permission of Vizient, Inc. All rights reserved. Derived using margins command post-estimation of GLM (see Supplemental Table 3 for full set of coefficients). 95% CI in parentheses.

eFigure 2. Adjusted Costs of Inpatient by ECMO/Mechanical Ventilation and Week

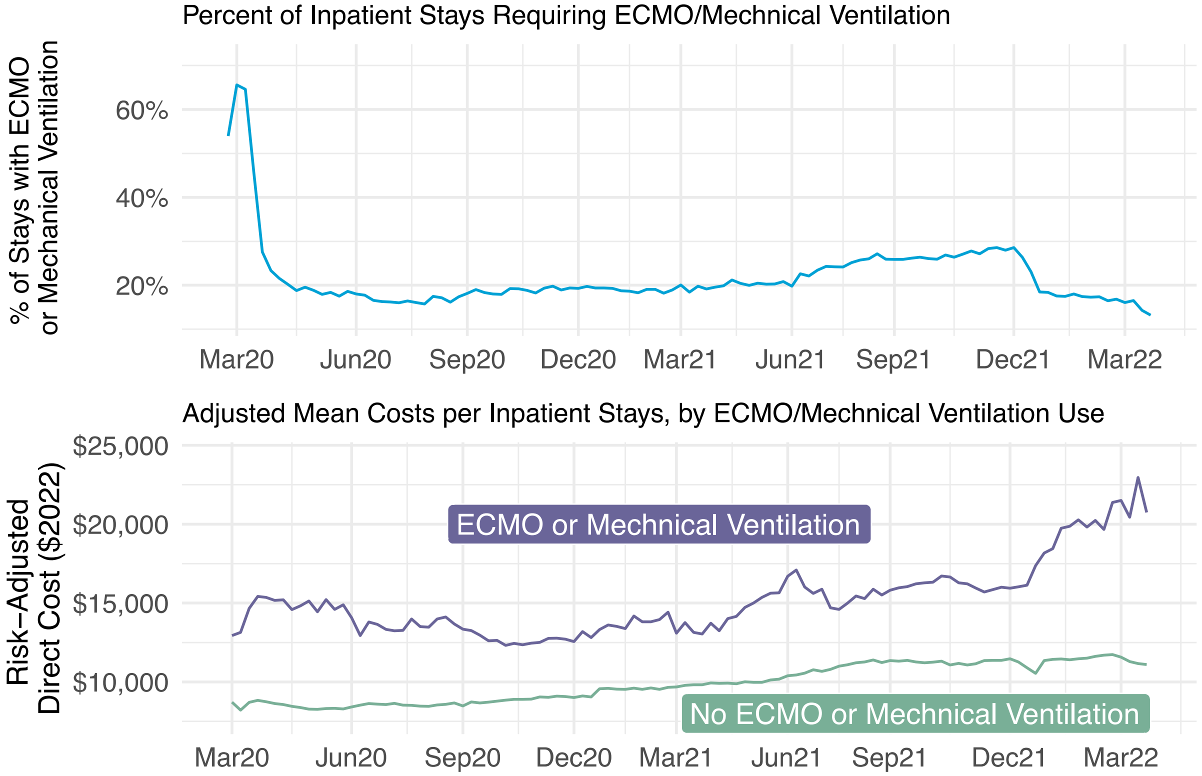

© 2024 Kapinos KA et al. *JAMA Network Open.*
