# Supplementary material for: Inpatient Costs of Treating Patients With COVID-19
Source: JAMA Netw Open. 2024 Jan 3;7(1):e2350145. doi: 10.1001/jamanetworkopen.2023.50145 (PMC10765267; doi:10.1001/jamanetworkopen.2023.50145)
Supplement: Supplement 2. — Data Sharing Statement [file jamanetwopen-e2350145-s002.pdf]

Data Sharing Statement

Kapinos. Inpatient Costs of Treating Patients With COVID-19. *JAMA Netw Open*. Published January 03, 2024. doi:10.1001/jamanetworkopen.2023.50145

Data
Data available: No

Additional Information
Explanation for why data not available: Proprietary data; those interested can apply to Vizient for access.
